# To Turn Inward or Outward? Examining the Reciprocal Relationships Between Mindfulness, Interpersonal Emotion Regulation, and Aggression Over Time

**DOI:** 10.21203/rs.3.rs-6985856/v1

**Published:** 2025-07-04

**Authors:** Erika Blair, David Chester

**Affiliations:** Virginia Commonwealth University; Virginia Commonwealth University

**Keywords:** mindfulness, interpersonal emotion regulation, aggression, emotion regulation, longitudinal study

## Abstract

People frequently turn to others to help regulate their emotions in what is referred to as *inter*personal emotion regulation (IER). Mindfulness entails an *intra*personal strategy of turning inward to facilitate emotion regulation. Yet little research has examined the relationships between these distinct regulation strategies and their consequences for aggression. The current study aims to elucidate how dispositional tendencies towards mindfulness and IER interact to predict each other and aggression over time. To do so, a diverse sample of undergraduates (*N* = 469) at a Minority Serving Institution completed a three-wave, longitudinal study with approximately 20 days between each wave. Against our predictions, between-participants estimates suggested that more mindful individuals engaged in less IER across time points. Paradoxically, within-participant analyses revealed that when participants were more mindful than usual, they subsequently engaged in *more* IER. IER and mindfulness did not consistently explain our measures of aggressive behavior. As both IER and mindfulness are effective regulatory approaches with salutary effects, the inverse relationship between the two raises important questions about the trade-offs between the costs and benefits associated with these approaches.

## Introduction

Emotion regulation is critical for healthy human functioning (Tamir, 2011). *Inter*personal emotion regulation (IER) has been linked to numerous positive emotional and social outcomes (Williams et al., 2018; Zaki & Williams, 2013), as has the more *intra*personal approach of mindfulness-based emotion regulation (Carpenter et al., 2019; Freudenthaler et al., 2017). A critical social outcome is aggressive behavior, which has been independently and negatively linked to mindfulness (Borders et al., 2010; Eisenlohr-Moul et al., 2016) and IER (Swerdlow et al., 2023). Yet no research to date has examined how IER and mindfulness might uniquely predict lower aggression. Further, studies on emotion-regulation and aggression are often cross-sectional and would benefit from the sophisticated modeling afforded by longitudinal design. To address these gaps in our understanding, we longitudinally investigated the predictive relationships between dispositional mindfulness and IER on aggression over time.

### Characteristics of IER

Whereas emotion regulation has been largely studied as an intrapersonal phenomenon, recent work has emphasized the importance of other people in the expression and regulation of emotions (Butler, 2015; Kappas, 2013). Indeed, people often turn to others to manage their emotional goals. IER refers to engagement in social interaction with the specific goal of influencing one’s own (intrinsic IER) or another’s (extrinsic IER) emotions (Dixon-Gordon et al., 2015; Williams et al., 2018; Zaki & Williams, 2013). Earlier research on the topic focuses on extrinsic IER, so the literature surrounding intrinsic IER is comparatively sparse (Williams et al., 2018). The regulation of one’s own internal emotional experience is of particular interest to the present study, so we focus exclusively on intrinsic IER. Intrinsic IER is further separated into two dimensions: *tendency* (i.e., how often someone turns to others to regulate their own emotions) and *efficacy* (i.e., how effective people perceive IER to be in achieving emotional goals) (Zaki & Williams, 2013). IER overlaps with constructs such as venting and support-seeking, but it represents a broader category of goal-directed social behavior specifically driven by the pursuit of shaping affective state (Niven, 2017; Williams, 2018).

#### IER in Daily Life.

IER is an important everyday method through which people pursue their emotional and other goals. On a weekly basis, people generally regulate their own emotions through others once a day (Tran et al., 2023a). Emotional goals during these interactions primarily center around increasing positive emotions (Tran et al., 2023a), although individuals frequently (i.e., about every other day over two weeks) modulate their mood by seeking emotional and problem-oriented support through sharing negative experiences (Liu et al., 2021). People also approach IER with instrumental goals outside of emotion regulation in mind. For instance, people engage in IER with the underlying motivation to seek clarity and pursue personal goals and build social relationships (Tran et al., 2024). People are more likely to share their experiences with close others (Liu et al., 2021), and individuals were less distressed after viewing negative stimuli after having a long-term relationship partner help regulate their emotions than when doing so alone (Levy-Gigi & Shamay-Tsoory, 2017). Thus, existing evidence suggests IER is integral to daily emotional goals and may be effective above-and-beyond intrapersonal emotion regulation in some cases. These findings also speak to the epistemic value of longitudinal studies of IER.

### Mindfulness and IER

Mindfulness is consistently associated with more effective *intra*personal emotion regulation (i.e., utilizing internal strategies to manage one’s emotions) (Carpenter et al., 2019; Freudenthaler et al., 2017; Hill & Updegraff, 2012; Roemer et al., 2015). Neural evidence suggests that mindful detachment may serve some emotion regulation benefits above other cognitive reappraisal strategies (Grecucci et al., 2015), and more mindful individuals recruit less neurobiological resources to regulate emotions (Lutz et al., 2014). Mindfulness was associated with more adaptive affective response to rejection which was mediated by less activation of top-down neural regulatory systems (Martelli et al., 2018). Therefore, evidence converges to suggest unique physiological mechanisms through which mindfulness promotes adaptive intrapersonal emotion regulation.

The emotional benefits of mindfulness are theorized to be attributed to an overall change in moment-to-moment experience. For example, an increased clarity of consciousness may allow one to disengage from habitual patterns of thought and behavior, allowing for greater self-regulation and enhanced quality of present experience (Brown & Ryan, 2003). Mindfulness is consistently associated with less experience of negative emotion (Roemer et al., 2015). This may also play a role in mindfulness’ demonstrated effectiveness at reducing negative thought patterns including rumination and catastrophizing (Tomlinson et al., 2018). The direct relationship between mindfulness and IER has yet to be studied. However, individuals with emotion regulation difficulties were more likely to turn to others to regulate their emotions (Malkoç et al., 2019). Further, individuals experiencing greater negative affect engaged in IER more often (Tran et al., 2023b). Therefore, some predictors of IER tendency (emotion dysregulation, negative affect) consistently display *negative* relationships with dispositional mindfulness.

### IER Outcomes

IER has been linked to numerous psychological outcomes. People who frequently employ IER display greater empathy, emotional expressiveness, and interpersonal competency (Malkoç et al., 2019; Niven & López-Pérez, 2025; Williams et al., 2018). IER may promote particularly salutary social outcomes, as it is consistently linked to the development of both a wider net and a greater quality of social connections (Niven & López-Pérez, 2025; Williams et al., 2018). Greater dependence on IER increased relationship satisfaction through more positive views of participants’ partners (Lemay et al., 2025). IER also facilitated social support among those managing depressive symptoms (Marroquín, 2011).

In some cases, IER may also provide benefits above and beyond intrapersonal emotion regulation. Socially reappraising distressing emotions was more effective both immediately and a day later compared to reappraising emotions alone (Sahi et al., 2025). IER may be of particular utility for individuals who experience difficulties with intrapersonal emotion regulation, as individuals who reported higher levels of emotional dysregulation still found IER to be a helpful avenue of emotion management (Swerdlow & Johnson, 2025). If IER functions to promote adaptive emotional and social outcomes such as improved emotion management and social support, then it likely also serves to mitigate maladaptive interpersonal outcomes such as aggressive behavior.

### Aggression and IER

Emotions drive aggression and as one might then expect, difficulties in *intra*personal emotion regulation are reliably linked to greater aggressive behavior (Ammerman et al., 2015). Yet little is known about the specific relationship between IER and aggression. People with clinically significant levels of aggression engaged in significantly less IER than controls, had more difficulty finding opportunities to engage in IER, and found IER to be less helpful (Swerdlow et al., 2023). Although this has not been investigated in a non-clinical sample, correlates of IER such as increased affective empathy and greater social support (Williams et al., 2018) have been negatively correlated with aggression (Kumar et al., 2014; Palumbo & Latzman, 2021). As such, there is good reason to expect that IER would be linked to less aggression.

### Mindfulness and Aggression

Mindfulness reduces both anger and aggression (Borders et al., 2010; Eisenlohr-Moul et al., 2016; Heppner et al., 2008), an effect that is mediated by reduced rumination and lower intensity of anger (Borders et al., 2010; Eisenlohr-Moul et al., 2016). Mindful individuals also display less intense anger and less engagement in retaliatory actions in response to injustice (Long & Christian, 2015). Mindfulness is characterized by the unique detachment from emotions and habitual thought patterns (Brown & Ryan, 2003; Grecucci et al., 2015), which may play a role in its negative association with maladaptive rumination (Raes & Williams, 2010; Tomlinson et al., 2018). Specifically, mindfulness may provide the benefit of non-judgmentally observing and effectively disengaging in ruminative responses, as demonstrated by its specific inverse relationship to *uncontrollable* rumination (Raes & Williams, 2010).

As rumination increases aggression (Bushman, 2005; Peled & Moretti, 2010), this is a key mechanism through which mindfulness reduces aggression.

### The Present Study

Most people engage in IER on a daily basis. Greater IER tendency and efficacy are linked to a host of positive social and psychological outcomes (Malkoç et al., 2019; Williams et al., 2018), underscoring its utility as an adaptive emotional behavior.

Mindfulness is also associated with positive socio-psychological outcomes, including more effective intrapersonal emotion regulation skills (Brown & Ryan, 2003; Roemer et al., 2015). There is reason to believe, however, that a *negative* relationship might exist between mindfulness and IER tendency. Individuals with more difficulties regulating emotions tend to turn to others to regulate their emotions through intrinsic IER (Malkoç et al., 2019). Because mindful individuals are particularly skilled at intrapersonal emotion regulation, we predicted that they would be less likely to engage in intrinsic IER.

Robust evidence links mindfulness to lower aggression (Borders et al., 2010; Eisenlohr-Moul et al., 2016; Long & Christian, 2015), and preliminary evidence suggests that IER tendency may also be negatively linked to aggression (Swerdlow et al., 2023). As both of these constructs are associated with less aggression, it is possible that the reduced tendency to engage in IER may actually disrupt the relationship between mindfulness and less aggression. Therefore, we proposed the following hypotheses:

1. Higher trait mindfulness will predict decreased aggression at the subsequent time point.
2. Higher trait mindfulness will predict individuals’ decreased tendency to seek others to regulate their emotions at the subsequent time point.
3. Greater tendency to seek others to regulate emotions will predict decreased aggression at the subsequent time point.
4. Greater tendency to seek others to regulate emotions will mediate the direct effect of mindfulness on aggression at the subsequent time point, such that decreased tendency to seek others to regulate emotions will suppress the negative effect of mindfulness on aggression.

## Method

### Transparency and Openness

We report all manipulations, measures, sample characteristics, sample size justification, and all data exclusions in the text. All analyses were conducted using *lavaan* R package (version 0.6–15; Rosseel, 2012). Data used in this analysis were previously collected at the institution in 2019 (data collection pre-registration plans: https://osf.io/m28gc; https://osf.io/ugyz5). We pre-registered the analytic plan and code for this secondary analysis (https://osf.io/ejzxy). De-identified data will be made publicly available (https://osf.io/tekba/).

### Participants

Participants were undergraduate students enrolled in an introductory psychology course at Virginia Commonwealth University. Initially, the sample consisted of 469 participants at wave 1. No *a priori* power analysis was conducted, but a sensitivity analysis run with the *semPower* package (version 0.12, Wang & Rhemtulla, 2021) revealed that the sample was large enough to detect a standardized regression path of *b* = .04 at 80% power at *α = .05*.

Eleven participants completed wave 2 and wave 3 within two days of each other. Six of these participants completed the surveys in the correct order; we removed these participants’ wave 3 data and replaced them with their wave 2 responses. The other five participants completed the wave 3 survey *before* wave 2. For these participants, we removed their wave 2 responses and retained their wave 3 data. After excluding these responses, the total sample size for wave 2 was 218 (46.5% of initial sample), and the total for wave 3 was 162 participants (34.5% of the initial sample). 22.4% of the participants completed all three waves (*n* = 105), 24.1% of the participants completed waves 1 and 2 but were missing wave 3 (*n* = 113), 12.2% of the participants completed waves 1 and 3 but were missing wave 2 (*n* = 57), and 41.4% of the participants only completed wave 1 (*n* = 194). Participants were compensated at each wave with course credit and a raffle ticket for a chance to win $25. Participant demographic information is displayed in Table 1.

### Materials

#### Mindful Attention and Awareness Scale.

Dispositional mindfulness was measured using the 15-item Mindful Awareness Attention Scale (MAAS; Brown & Ryan, 2003). The participants indicated how frequently they experienced the described experience (e.g., “I could be experiencing some emotion and not be conscious of it until some time later”), using a 6-point Likert scale from 1 (*almost always*) to 6 (*almost never*). We reversed all scores and averaged each item to create a total mindfulness score, with higher scores indicating greater mindfulness.

#### Interpersonal Regulation Questionnaire.

Interpersonal emotion regulation was measured using the 16-item Interpersonal Regulation Questionnaire (IRQ; Williams et al., 2018). Participants indicated the degree to which they agreed with each statement on a scale from 1 (*strongly disagree*) to 7 (*strongly agree*). The IRQ is divided into two subscales (positive tendency, negative tendency, positive efficacy, and negative efficacy). Although participants completed all questions, we only analyzed the responses to positive tendency questions (e.g., “when things are going well, I just have to tell other people about it”) and negative tendency questions (e.g., “I just have to get help from someone when things are going wrong”) for a total of eight responses. We averaged the responses to all tendency questions to calculate a total IER tendency score, with higher scores indicating a greater tendency to regulate emotions socially.

#### Doll Aggression Task.

Participants completed an online version of The Doll Aggression Task (DAT; DeWall et al., 2013) to assess aggressive behavior. Participants were first instructed to *““Please take a moment and imagine a real person from your life that you feel very angry towards”*. Participants then viewed an image of a burlap doll and were instructed to imagine the doll as the person they feel angry towards. The screen then displayed the doll with 51 pins stuck into it, then with no pins. The participants were then instructed to use a slider to select a number of pins, on a scale from 0 to 51, they would like to stick into the doll. We calculated final DAT scores by adding one to the raw scores (i.e., the number of pins the participants chose) and applying a base 10 logarithmic transformation to reduce positive skew and kurtosis (Chester & Lasko, 2019). Final DAT scores represented aggressive behavior, with higher scores indicating greater aggression.

#### The Buss-Perry Aggression Questionnaire.

The 9-item Physical Aggression subscale of the Buss-Perry Aggression Questionnaire (BPAQ; Buss & Perry, 1992) measured participants’ dispositional aggression. Participants rated how well each statement described them (e.g., “Once in a while, I can’t control the urge to strike another person.”) on a scale of 1 (*extremely uncharacteristic)* to 7 (*extremely characteristic*). We averaged participants’ scores from each item to calculate a total dispositional aggression score, with higher scores indicating greater aggression.

#### The Five-Factor Mindfulness Questionnaire.

Participants responded to the 39-item Five Factor Mindfulness Questionnaire (FFMQ; Baer et al., 2006) as another measure of dispositional mindfulness. The FFMQ is comprised of five subscales: observing, describing, acting with awareness, nonjudging, and nonreactivity. Participants rated how much they agreed with each item (e.g., “I pay attention to how my emotions affect my thoughts and behavior”) on a scale from 1 (*never or very rarely true*) to 5 (*very often or always true*). Items were appropriately reverse-coded and averaged to create a total dispositional mindfulness score, with a higher score indicating greater mindfulness.

### Procedure

The participants completed all three waves of the study online. The procedures for each wave were identical. Participants received a link to an online survey about the influences of personality on behaviors, and they were instructed to complete the survey on their own time. After accessing the survey and consenting to participate, participants completed the MAAS, DAT, FFMQ, IRQ, and BPAQ. After completing the first wave, participants received an interim debriefing and were asked to give their contact information. We emailed participants with the link for the second wave two weeks (14 days) after the first, and we emailed the participants the link for the third wave four weeks (28 days) after the completion of the first wave.

### Analytic Plan

We conducted two random intercept cross-lag panel models (RI-CLPMs) using *lavaan* R package (version 0.6–15; Rosseel, 2012). RI-CLPMs are extensions of traditional longitudinal cross-lagged panel models in that they model the effect of one predictor at one time point on an outcome at a subsequent time point. Critically, RI-CLPMs also control for stable trait-like variables, thus allowing for the elucidation of both within-participant and between-participant effects (Lüdtke & Robitzsch, 2021). We selected this framework in order to analyze the potential directional and reciprocal within-participant effects of mindfulness, IER tendency, and aggression over time while accounting for variation between participants. Cross-lagged regression estimates between waves, latent intercepts across waves, and mediation pathways are displayed in the results tables (Tables 2 and 3). The models also estimated autoregressive effects and within-wave covariances (see Supplemental Materials). Missing data was handled via FIML.

## Results

### Time Between Waves

Participants completed the second wave 12–49 days after the first wave, *M* = 18.39 days, *SD* = 5.96, mode = 14 days (24.9% of the wave 2 sample with this modal timeframe). Participants completed the third wave 13–44 days after the second wave, *M* = 20.50 days, *SD* = 8.12, modes = 14 and 15 days (34.2% of the wave 3 sample with this modal timeframe). Participants completed the third wave 26–63 days after the first wave, *M* = 40.21 days, *SD* = 9.35, mode = 33 days (7.5% of the wave 3 sample with this modal timeframe).

### Descriptive Statistics

We calculated all descriptive and internal consistency statistics using SPSS (Version 29) and are displayed in Table 1.

### Confirmatory Cross-Lag Models

#### RI-CLPM 1: Lagged effects of mindfulness, interpersonal emotion regulation, and aggressive behavior.

RI-CLPM 1 fit was acceptable, *χ*^2^ = 18.59, *p* = .099, *RMSEA* = .03 (*90% CI* = .00, .06), *SRMR* = .04, *TLI* = .98, *CFI* = .99. Within-participant cross-lagged paths were non-significant, except dispositional mindfulness predicted IER in the following wave (but not vice versa). Between-participants, however, the opposite effect was observed in which mindfulness was negatively associated with IER. Mindfulness and aggression were also negatively associated between-participants. Contrary to our pre-registered hypotheses, no significant mediation pathway between mindfulness, IER, and aggressive behavior was observed (Table 2).

#### RI-CLPM 2: Lagged effects of mindfulness, interpersonal emotion regulation, and dispositional aggression.

RI-CLPM 2 fit was acceptable, *χ*^2^ = 15.06, *p* = .238, *RMSEA* = .02 (*90% CI* = .00, .05), *SRMR* = .04, *TLI* = .99, *CFI* = .99. The results of Model 2 mirrored those of Model 1. Within-participant cross-lagged paths were non-significant, except dispositional mindfulness predicted IER in the following wave (but not vice versa). Between-participants, mindfulness was negatively associated with both IER and aggression. Contrary to our pre-registered hypotheses, no significant mediation pathway between mindfulness, IER, and dispositional aggression was observed (Table 3).

### Exploratory Robustness Analyses Using a Different, Broader Measure of Mindfulness

We ran two more RI-CLPMs to investigate whether the effects found in Models 1 and 2 would replicate using a different, multi-faceted measure of a broader conceptualization of mindfulness (i.e., the total score of the FFMQ). These models were not pre-registered and therefore serve as exploratory analyses.

#### RI-CLPM 3: Lagged effects of five-facet mindfulness, interpersonal emotion regulation, and aggressive behavior.

RI-CLPM 3 fit was acceptable, *χ*^2^ = 19.22, *p* = .083, *RMSEA* = .04 (*90% CI* = .00, .06), *SRMR* = .03, *TLI* = .98, *CFI* = .99. No cross-lagged paths were significant. Consistent with RI-CLPMs 1 and 2, between-participant mindfulness was negatively associated with both IER and aggression. In this model, an additional negative association between IER and aggression was also observed. No significant mediation pathway between mindfulness, IER, and aggressive behavior was observed (Table 4).

#### RI-CLPM 4: Lagged effects of five-facet mindfulness, interpersonal emotion regulation, and dispositional aggression.

RI-CLPM 4 fit was acceptable, *χ*^2^ = 12.68, *p* = .393, *RMSEA* = .01 (*90% CI* = .00, .05), *SRMR* = .03, *TLI* = .99, *CFI* = .99. No cross-lagged paths were significant. Consistent with RI-CLPMs 1, 2, and 3, mindfulness was negatively associated with IER between-participants. Consistent with RI-CLPM 3, a negative association between IER and aggression was also observed. No significant mediation pathway between mindfulness, IER, and dispositional aggression was observed (Table 5).

### Exploratory Robustness Analyses Examining the Efficacy of Interpersonal Emotion Regulation

We ran RICLPM 1 and RICLPM 2 again using the efficacy subscale of the IRQ instead of the tendency subscale as additional exploratory analyses (Models 5 and 6, see Supplemental Materials). Critically, while mindfulness and IER tendency consistently showed a negative between-participant association across all tendency models, this effect became statistically non-significant when examining IER efficacy.

## Discussion

Emotion regulation is a key component of overall emotional experience (Tamir, 2011). It is essential to study emotion regulation in a social context, as people frequently turn to others to regulate their emotions (Liu et al., 2021; Tran et al., 2023a). Both mindfulness and IER are related to positive psychological outcomes through adaptive emotion regulation and lower aggression (Brown & Ryan, 2003; Swerdlow et al., 2023). However, the literature surrounding intrinsic IER is sparse and inconclusive regarding its social-psychological outcomes (Zaki, 2018), and no study has investigated the interplay between mindfulness, IER, and aggression. The current study aimed to elucidate these relationships, and we hypothesized that a) mindfulness would predict reduced aggression over time, b) mindfulness would predict reduced IER tendency over time, c) IER tendency would predict reduced aggression over time, and d) reduced IER tendency would exhibit a suppression effect on the relationship between mindfulness and aggression, reducing the strength of the negative relationship.

Our confirmatory models did not support our pre-registered hypotheses. Both models only displayed one significant cross-lagged pathway, in which mindfulness predicted greater IER in the following waves. In other words, when individuals were more mindful than normal, they displayed more IER tendency at a later time point than they usually do. This finding ran counter to our hypotheses. Notably, however, IER tendency and dispositional mindfulness were *negatively* associated between participants across waves. In other words, overall, more mindful individuals exhibited less tendency to turn to other people to regulate their emotions. This was in line with our predictions that more mindful people would engage in less IER. Further, this finding was replicated in exploratory models, demonstrating the robustness of this effect across aggression and mindfulness measures.

The seemingly contradictory within and between-participant results are an example of Simpson’s Paradox (Simpson, 1951). Simpson’s Paradox refers to the phenomenon in which statistical trends exhibited at individual levels are nonexistent or reversed when the data is analyzed at an aggregate level. Although it appears counterintuitive, Simpson’s Paradox is not an anomaly and is widely recognized in psychological science (Kievet et al., 2013). With stable, trait-level variables, sometimes trends that manifest at the aggregate level are not consistent with how individuals change over time. For example, Shulman et al. (2024) examined the cross-lagged relationships between prejudice and contact with ethnic minorities over time. While they found that people who experienced more contact were also less prejudiced, fluctuations in contact did not predict fluctuations in prejudice over time. That is, at times when individuals experienced more contact with ethnic minorities than usual, this was not followed by reduced prejudice in the future, suggesting that individual events of contact do not causally shift prejudice (Shulman et al., 2024). In another example, neurotic individuals may be less likely to drink coffee even though drinking coffee increases neuroticism. This could be explained by more neurotic individuals displaying greater health concern and therefore drinking less coffee, yet coffee still predicting increases in neuroticism for all individuals (Kievet et al., 2013).

Mindfulness, as measured in the present study, is a stable, trait-like variable (Baer et al., 2006; Brown & Ryan, 2003). Therefore, individual fluctuations over time may not reflect the same trends observed across people. Our between-participant results were consistent with the evidence that people with emotion regulation difficulties tend to turn to others more when regulating emotions (Malkoç, 2019), and more mindful people are more skilled at emotion regulation (Roemer et al., 2015). Thus, it is logical that more mindful individuals turn more often to intrapersonal vs. interpersonal emotion regulation strategies to pursue emotional goals. Exploratory analyses further underscored this logic by showing that this relationship was specific to IER tendency and not efficacy. However, when individuals are less mindful than usual, they may be less likely to regulate their emotions through IER. This might not reflect a tendency to turn to other emotion regulation strategies, but instead reflect a *general* reduction in emotion regulation behaviors. Mindfulness can buffer avoidant reactions to emotional distress (Colombo et al., 2023). When people are feeling less mindful than usual, it is possible they are more likely to avoid rather than approach their emotions.

The results of this study provided important evidence about the complexity of interpersonal emotion regulation and its relationship to mindfulness. Particularly, results displayed how individual differences in these characteristics might not reflect how they change over time. The most consistent finding, however, was the negative association between mindfulness and IER tendency. This has implications for the role of mindfulness in the developing field of emotion regulation. Both mindfulness and IER display adaptive psychological outcomes (Brown & Ryan, 2003; Roemer et al., 2015; Williams et al., 2018), but they are negatively associated with each other. This raises questions about the nuanced functions of both variables independently as well as their interactive effects in promoting (or hindering) adaptive emotion regulation goals.

### Limitations and Future Directions

The results of this study may have been biased by participant attrition rates. The substantial loss of participants between waves potentially skewed the results through non-random missing data. Future studies with greater retention rates would be valuable in determining whether or not these results replicate. Further, the sample consisted of only undergraduate university students. A more diverse sample across a variety of age ranges would provide important information about the generalizability of the results.

The study measured IER in terms of tendency and efficacy. However, we did not examine specific strategies of emotion regulation within IER. Evidence shows that people pursue emotion regulation through a variety of distinct strategies, such as through reappraisal, problem-solving, rumination, acceptance, and suppression (Kneeland et al., 2024). As the relationship between mindfulness and IER is complex, future studies could benefit from examining how these strategies interact differently as they apply to mindfulness and overall psychological outcomes. Additionally, the emotional goals of IER were only centered around reducing negative emotion and increasing positive emotion. Future studies could investigate these relationships in the context of the reverse goals; that is, turning to others to increase negative emotions or decrease positive emotions.

## Conclusion

Social interactions are integral to our emotional lives, and we often turn to others to help us regulate our emotions. Despite the ubiquity of this behavior, knowledge about the process and outcomes of intrinsic interpersonal emotion regulation is still developing. This study supported the tendency for more mindful individuals to engage in less IER. Both mindfulness and IER are associated with positive social-psychological outcomes, so the results raise important questions about the complexity of these constructs as they relate to adaptive emotional functioning. Applications of mindfulness interventions to social outcomes might benefit from the knowledge that more mindful individuals are less likely to engage in emotion regulation with the help of others. While the results of this study did not provide evidence of this phenomena with regards to aggression outcomes, they may carry implications for other positive correlates of IER such as social connection and interpersonal competence.

## Supplementary Files

This is a list of supplementary files associated with this preprint. Click to download.

- SupplementalMaterialsMindfulnessIERAggression.pdf

## Figures and Tables

**Table 1:** Participants Demographics by Wave

Demographics	Wave 1 (N = 469)	Wave 2 (N = 218)	Wave 3 (N = 162)
Age			
M (SD)	18.90 (2.43)	19.19 (2.70)	18.79 (1.48)
Range	18 – 49	18 – 49	18 – 28
Missing: N (%)	8 (1.7%)	8 (3.7%)	4 (2.5%)
Gender: N (%)			
Cisgender Female	342 (73.0%)	165 (75.7%)	124 (76.5%)
Cisgender Male	124 (26.4%)	50 (22.9%)	36 (22.2%)
Transgender Male	1 (0.2%)	1 (0.5%)	1 (0.6%)
+	2 (0.4%)	2 (0.9%)	1 (0.6%)
Missing	0 (0.0%)	0 (0.0%)	0 (0.0%)
Race/Ethnicity: N (%)			
White / Caucasian	199 (42.4%)	101 (46.3%)	68 (42.0%)
Black / African-American	100 (21.4%)	33 (15.1%)	25 (15.4%)
Hispanic / Latino(a)	44 (9.4%)	25 (11.5%)	18 (11.1%)
Native American	1 (0.2%)	0 (0.0%)	0 (0.0%)
Asian	74 (15.8%)	39 (17.9%)	30 (18.5%)
+	49 (10.4%)	19 (8.7%)	20 (12.3%)
Missing	2 (0.4%)	1 (0.5%)	1 (0.6%)
Sexual Orientation			
Heterosexual	388 (82.7%)	184 (80.3%)	125 (77.2%)
Gay	8 (1.7%)	6 (2.6%)	4 (2.5%)
Lesbian	10 (2.1%)	4 (1.7%)	3 (1.9%)
Bisexual	47 (10.1%)	29 (12.7%)	23 (14.6%)
Asexual	2 (0.4%)	0 (0.0%)	0 (0.0%)
+	9 (1.9%)	3 (1.3%)	3 (1.9%)
Missing	5 (1.1%)	3 (1.3%)	4 (2.5%)
Household Income: N (%)			
<$25,000	42 (9.0%)	24 (10.5%)	16 (9.9%)
$25,000 - $39,999	57 (12.2%)	21 (9.2%)	19 (11.7%)
$40,000 - $54,999	48 (10.2%)	27 (11.8%)	20 (12.3%)
$55,000 - $69,999	35 (7.5%)	20 (8.7%)	15 (9.3%)
$70,000 - $159,999	199 (42.4%)	98 (42.9%)	60 (37.0%)
$160,000 or more	82 (17.4%)	36 (15.7%)	30 (18.5%)
Missing	6 (1.3%)	3 (1.3%)	2 (1.2%)

*Note*. + = identity was not listed. Race/ethnicity was measured in mutually exclusive categories

**Table 1:** Descriptive Statistics

Measure	Wave	*M*	*SD*	Range	*N*	*α*
MAAS	1	3.57	0.86	1.33–6.00	467	.89
	2	3.66	0.89	1.00–6.00	217	.92
	3	3.73	0.94	1.47–6.00	160	.93
DAT	1	15.65	16.80	0.00–51.00	460	
	2	14.17	15.92	0.00–51.00	209	
	3	12.83	15.44	0.00–51.00	150	
BPAQPA	1	2.80	1.29	1.00–6.44	458	.89
	2	2.62	1.32	1.00–7.00	214	.83
	3	2.58	1.28	1.00–6.11	159	.81
IRQ	1	4.62	1.16	1.00–7.00	460	.81
	2	4.72	1.15	1.00–7.00	214	.87
	3	4.80	1.16	1.00–7.00	159	.87
FFMQ	1	3.10	0.45	1.44–4.72	460	.89
	2	3.09	0.46	1.90–5.00	214	.89
	3	3.11	0.47	1.55–4.67	159	.90

**Table 2:** Parameter Estimates from Random-Intercept Cross-Lagged Panel Model 1

Model Parameters		*β*	*B*	*SE*	*Z*	*p*
Cross-Lagged Paths						
W1: MAAS ◊	W2: DAT	0.12	0.05	0.07	0.68	.495
W2: MAAS ◊	W3: DAT	0.09				
W1: DAT ◊	W2: MAAS	−0.17	−0.20	0.20	−0.98	.329
W2: DAT ◊	W3: MAAS	−0.06				
**W1: MAAS ◊**	**W2: IRQ**	**0.33**	**0.44**	**0.16**	**2.85**	**.004**
**W2: MAAS ◊**	**W3: IRQ**	**0.31**				
W1: IRQ ◊	W2: MAAS	0.25	0.18	0.12	1.50	.133
W2: IRQ ◊	W3: MAAS	0.19				
W1: IRQ ◊	W2: DAT	−0.37	−0.11	0.08	−1.41	.159
W2: IRQ ◊	W3: DAT	−0.26				
W1: DAT ◊	W2: IRQ	0.10	0.17	0.27	0.63	.530
W2: DAT ◊	W3: IRQ	0.05				
Across-Wave Covariance						
**RI MAAS**	**RI DAT**	**−0.28**	**−0.11**	**0.03**	**−4.16**	**< .001**
**RI MAAS**	**RI IRQ**	**−0.25**	**−0.18**	**0.05**	**−3.63**	**< .001**
RI IRQ	RI DAT	0.09	0.05	0.04	1.30	.194
Indirect and Total Effects						
Indirect		−0.12	−0.05	0.04	−1.31	.191
Total		0.002	0.001	0.08	0.02	.986

*Note*. W1 = Wave 1; W2 = Wave 2; W3 = Wave 3; RI = Random Intercept; MAAS = Mindful Attention and Awareness Scale; DAT = Doll Aggression Task - Log Transformed; IRQ = Interpersonal Emotion Regulation – Tendency Subscale. Bolded values are significant at *p* < .05.

**Table 3:** Parameter Estimates from Random-Intercept Cross-Lagged Panel Model 2

Model Parameters		*β*	*B*	*SE*	*Z*	*p*
Cross-Lagged Paths						
W1: MAAS ◊	W2: BPAQPA	0.16	0.28	0.22	1.28	.200
W2: MAAS ◊	W3: BPAQPA	0.23				
W1: BPAQPA ◊	W2: MAAS	−0.09	−0.06	0.13	−0.45	.655
W2: BPAQPA ◊	W3: MAAS	−0.09				
**W1: MAAS ◊**	**W2: IRQ**	**0.30**	**0.41**	**0.16**	**2.60**	**.009**
**W2: MAAS ◊**	**W3: IRQ**	**0.29**				
W1: IRQ ◊	W2: MAAS	0.16	0.12	0.14	0.87	.384
W2: IRQ ◊	W3: MAAS	0.13				
W1: IRQ ◊	W2: BPAQPA	0.01	0.01	0.21	0.06	.949
W2: IRQ ◊	W3: BPAQPA	0.02				
W1: BPAQPA ◊	W2: IRQ	−0.01	−0.01	0.15	−0.08	.938
W2: BPAQPA ◊	W3: IRQ	−0.02				
Across-Wave Covariance						
**RI MAAS**	**RI BPAQPA**	**−0.21**	**−0.16**	**0.06**	**−2.87**	**.004**
**RI MAAS**	**RI IRQ**	**−0.22**	**−0.16**	**0.05**	**−3.21**	**.001**
**RI IRQ**	**RI BPAQPA**	**−0.19**	**−0.20**	**0.08**	**−2.34**	**.019**
Indirect and Total Effects						
Indirect		0.003	0.05	0.09	0.06	.949
Total		0.16	0.29	0.18	1.60	.111

*Note*. W1 = Wave 1; W2 = Wave 2; W3 = Wave 3; RI = Random Intercept; MAAS = Mindful Attention and Awareness Scale; Buss-Perry Aggression Questionnaire-Physical Aggression Subscale; IRQ = Interpersonal Emotion Regulation – Tendency Subscale. Bolded values are significant at *p* < .05.

**Table 4:** Parameter Estimates from Random-Intercept Cross-Lagged Panel Model 3

Model Parameters		*β*	*B*	*SE*	*Z*	*p*
Cross-Lagged Paths						
W1: FFMQ ◊	W2: DAT	−0.24	−0.24	0.21	−1.18	.239
W2: FFMQ ◊	W3: DAT	−0.19				
W1: DAT ◊	W2: FFMQ	−0.28	−0.17	0.11	−1.53	.127
W2: DAT ◊	W3: FFMQ	−0.15				
W1: FFMQ ◊	W2: IRQ	0.15	0.49	0.38	1.30	.195
W2: FFMQ ◊	W3: IRQ	0.18				
W1: IRQ ◊	W2: FFMQ	0.22	0.08	0.06	1.41	.160
W2: IRQ ◊	W3: FFMQ	0.23				
W1: IRQ ◊	W2: DAT	−0.37	−0.12	0.10	−1.25	.210
W2: IRQ ◊	W3: DAT	−0.28				
W1: DAT ◊	W2: IRQ	−0.05	−0.09	0.33	−0.26	.793
W2: DAT ◊	W3: IRQ	−0.03				
Across-Wave Covariance						
**RI FFMQ**	**RI DAT**	**−0.19**	**−0.04**	**0.02**	**−2.48**	**.013**
**RI FFMQ**	**RI IRQ**	**−0.18**	**−0.07**	**0.03**	**−2.54**	**.011**
RI IRQ	RI DAT	0.14	0.07	0.05	1.51	.130
Indirect and Total Effects						
Indirect		−0.06	−0.06	0.06	−0.99	.321
Total		−0.30	−0.30	0.21	−1.40	.161

*Note*. W1 = Wave 1; W2 = Wave 2; W3 = Wave 3; RI = Random Intercept; FFMQ = Five Factor Mindfulness Questionnaire; DAT = Doll Aggression Task - Log Transformed; IRQ = Interpersonal Emotion Regulation –Tendency Subscale. Bolded values are significant at *p* < .05.

**Table 5:** Parameter Estimates from Random-Intercept Cross-Lagged Panel Model 4

Model Parameters		*β*	*B*	*SE*	*Z*	*p*
Cross-Lagged Paths						
W1: FFMQ →	W2: BPAQPA	−0.14	−0.49	0.47	−1.05	.293
W2: FFMQ →	W3: BPAQPA	−0.19				
W1: BPAQPA →	W2: FFMQ	−0.26	−0.08	0.06	−1.47	.142
W2: BPAQPA	W3: FFMQ	−0.23				
W1: FFMQ →	W2: IRQ	0.18	0.65	0.43	1.52	.128
W2: FFMQ →	W3: IRQ	0.21				
W1: IRQ →	W2: FFMQ	0.22	0.07	0.06	1.24	.214
W2: IRQ →	W3: FFMQ	0.22				
W1: IRQ →	W2: BPAQPA	0.21	0.22	0.17	1.27	.203
W2: IRQ →	W3: BPAQPA	0.28				
W1: BPAQPA →	W2: IRQ	0.17	0.17	0.15	1.12	.261
W2: BPAQPA →	W3: IRQ	0.18				
Across-Wave Covariance						
RI FFMQ	RI BPAQPA	−0.08	−0.03	0.03	−1.18	.239
**RI FFMQ**	**RI IRQ**	**−0.17**	**−0.07**	**0.03**	**−2.47**	**.013**
**RI IRQ**	**RI BPAQPA**	**−0.22**	**−0.23**	**0.09**	**−2.64**	**.008**
Indirect and Total Effects						
Indirect		0.04	0.14	0.18	0.81	.418
Total		−0.10	−0.35	0.54	−0.64	.524

*Note*. W1 = Wave 1; W2 = Wave 2; W3 = Wave 3; RI = Random Intercept; FFMQ = Five Factor Mindfulness Questionnaire; Buss-Perry Aggression Questionnaire-Physical
